# Lovastatin Production by *Aspergillus sclerotiorum* Using Agricultural Waste

**DOI:** 10.17113/ftb.58.02.20.6223

**Published:** 2020-06

**Authors:** Jutarut Iewkittayakorn, Kannika Kuechoo, Yaowapa Sukpondma, Vatcharin Rukachaisirikul, Souwalak Phongpaichit, Wilaiwan Chotigeat

**Affiliations:** 1 Department of Molecular Biotechnology and Bioinformatics, Faculty of Science, Prince of Songkla University, Hat Yai, 90112 Songkhla, Thailand; 2 Department of Chemistry and Center of Excellence for Innovation in Chemistry, Faculty of Science, Prince of Songkla University, Hat Yai, 90112 Songkhla, Thailand; 3 Department of Microbiology, Faculty of Science, Prince of Songkla University, Hat Yai, 90112 Songkhla, Thailand; 4 Center for Genomics and Bioinformatics Research, Faculty of Science, Prince of Songkla University, Hat Yai, 90112 Songkhla, Thailand

**Keywords:** agricultural waste, *Aspergillus sclerotiorum*, lovastatin, solid-state fermentation, soya bean sludge

## Abstract

**Research background:**

Lovastatin is a well-known drug used to reduce hypercholesterolaemia. However, the cost of lovastatin production is still high. Therefore, alternative low-cost carbon sources for the production of lovastatin are desirable.

**Experimental approach:**

Four different agricultural wastes, namely corn trunks, rice husks, wild sugarcane, and soya bean sludge, were tested separately as substrates to produce lovastatin using a new fungal strain, *Aspergillus sclerotiorum* PSU-RSPG 178, under both submerged and solid-state fermentation (SSF).

**Results and conclusions:**

Of these substrates and cultivation systems, soya bean sludge gave the highest lovastatin yield on dry mass basis of 0.04 mg/g after 14 days of SSF at 25 °C. Therefore, the soya bean sludge was separately supplemented with glucose, wheat flour, trace elements, palm oil, urea and molasses. The addition of the palm oil enhanced the lovastatin yield to 0.99 mg/g. In addition, the optimum conditions, which gave a lovastatin yield of (20±2) mg/g after 18 days of SSF, were soya bean sludge containing 80% moisture (dry basis) at a ratio of soya bean sludge (g) to mycelial agar plugs of 1:4, and a ratio of soya bean sludge (g) to palm oil (mL) of 1:2. Besides, the lovastatin yields obtained from SSF using fresh or dry soya bean sludge were not significantly different.

**Novelty and scientific contribution:**

We conclude that *A. sclerotiorum* PSU-RSPG 178 has a good potential as an alternative strain for producing lovastatin using soya bean sludge supplemented with palm oil as a carbon source.

## INTRODUCTION

Lovastatin (C_24_H_36_O_5_) is a potent drug for lowering the blood cholesterol level of humans and animals. It inhibits 3-hydroxy-3-methylglutaryl-CoA (HMG-CoA) reductase, which is a rate**-**limiting enzyme in cholesterol biosynthesis (*1*). Lovastatin is a natural statin that can be produced through microbial fermentation as a secondary metabolite (*2*). Several fungal species are good lovastatin producers, especially *Aspergillus terreus, Doratomyces stemonitis, Eupenicillium javanicum, Monascus pilosus, M. purpureus, M. ruber* and *Penicillium* spp. (*3*).

Both submerged fermentation (SmF) and solid-state fermentation (SSF) have been used for lovastatin production (*4*). SmF is a process of cultivation of microorganisms in broth (aqueous phase), while SSF is applied for cultivation of microorganisms in solid substrates without a free-flowing aqueous phase (*5*). SmF has been successful in the industrial production of lovastatin using *A. terreus.* In the laboratory, *A. terreus* ATCC 20542 (commercial strain) cultured in a lactose-based medium under SmF at 28 °C and shaken at 200 rpm yielded 873 mg**/**L of lovastatin on day 10 (*6*), while the same strain cultured in a mixture of glycerol and lactose under SmF at 30 °C yielded only 122 mg**/**L on day 7 (*7*). The SSF process has attracted much research interest due to its many advantages over conventional SmF, such as higher yields of secondary metabolites and enzymes (*8*). SSF can be used with agricultural residues and it has lower energy requirements (*9*, *10*). Agricultural raw materials that have been used for lovastatin production under SSF include corn, rice and sorghum grain. However, these materials are not only expensive, but their use as a raw material also competes with their role as food or animal feed. Therefore, efforts to reduce the cost of lovastatin production have been mainly directed at finding a new substrate that is readily available and sufficiently cheap. Large quantities of agricultural waste are produced after crop harvesting each year and cause a lot of environmental problems. These wastes can be used as substrates for the production of lovastatin by microorganisms. For example, Ruchir and Rekha (*11*) used wheat bran, corn hull and rice husk to produce lovastatin with *Aspergillus terreus* UV 1617; and wheat bran supported the highest production. Jahromi *et al.* (*12*), using rice straw in SSF, obtained maximal lovastatin levels of 0.18 and 0.26 mg**/**g with *A. terreus* ATCC 20542 and ATCC 74135, respectively.

Phainuphong *et al*. (*13*) produced lovastatin from a new strain, *A*. *sclerotiorum* PSU-RSPG 178, which was isolated from a soil sample collected from the Plant Genetic Conservation Project under the Royal Initiation of Her Royal Highness Princess Maha Chakri Sirindhorn at Ratchaprapa Dam in Suratthani Province, Thailand. This strain produced a high yield of lovastatin, 1316 mg**/**L, in nutrient broth medium (*14*) and therefore seems to have good potential for lovastatin production.

This research aims to optimize lovastatin production from *A. sclerotiorum* PSU-RSPG 178 under SmF and SSF using agricultural wastes. The agricultural wastes used as substrates were corn trunks, rice husks, wild sugarcane and soya bean sludge. The substrate that gave the highest lovastatin yield was further supplemented with glucose, wheat flour, trace elements, palm oil, urea and molasses. In addition, other fermentation parameters were investigated: the ratios of medium (g) to mycelial agar plugs of 1:2 to 1:6, ratios of medium (g) to supplement source (mL) of 1:2 to 1:6, and fermentation times of 14 to 21 days.

## MATERIALS AND METHODS

### Microorganism and inoculum preparation

*Aspergillus sclerotiorum* PSU-RSPG178 was isolated from a soil sample collected from the Plant Genetic Conservation Project under the Royal Initiation of Her Royal Highness Princess Maha Chakri Sirindhorn at Ratchaprapa Dam in Suratthani Province, Thailand. It was grown on potato dextrose agar (PDA) which was obtained from HiMedia, Mumbai, India at 28 °C for 7 days. Mycelial plugs (0.5 cm×0.5 cm) cut from the periphery of an actively growing colony were used as the inoculum according to the method of Daengrot *et al*. (*15*). The mycelial plugs were used since a spore inoculum gave a low yield of lovastatin in a previous study (*14*).

### Substrates and chemicals

The agricultural wastes used in this study were corn trunks, rice husks, wild sugarcane (*Saccharum spontaneum*) and soya bean sludge. The corn trunks, rice husks, and wild sugarcane were obtained from farmers in Khuan Khanun, Phatthalung, Thailand, while fresh soya bean sludge was obtained from a tofu shop in Hat Yai, Songkhla, Thailand. The corn trunks and wild sugarcane were washed with water and cut to a length of 0.5 cm. All the wastes were dried at 60 °C to a constant mass. Wheat flour, molasses and palm oil (Morakot, olein palm oil) were obtained from a local market in Hat Yai, Songkhla, Thailand. All chemicals were of analytical grade, including glucose (d-glucose anhydrous, Kemaus, Cherybrook, NSW, Australia), urea (99.5%, Kemaus), KH_2_PO_4_ (anhydrous, Loba Chemie, Mumbai, India), MgSO_4_·7H_2_O, CaCl_2_ (dihydrate, FeSO_4_·7H_2_O and ZnSO_4_·7H_2_O (Fisher Scientific, Leicestershire, UK), and methanol, hexane, ethyl acetate, Na_2_SO_4_ and acetonitrile (all RCI Labscan, HPLC grade, Bangkok, Thailand).

### Culture systems and conditions

For SmF, 5 g (dry mass) samples of waste were placed in 250-mL Erlenmeyer flasks to which 100 mL of distilled water were added. The mixture was autoclaved for 15 min at 103 kPa and 121 °C, then cooled to room temperature. Five mycelial agar plugs (0.5 cm×0.5 cm) of *A. sclerotiorum* PSU-RSPG178 were added to each Erlenmeyer flask. The flasks were incubated at 25 °C for 14 days. The solid waste that gave the highest yield of lovastatin concentration was selected to be cultured under SSF.

For SSF, 5 g (dry mass) samples of the selected solid waste were placed in 250-mL Erlenmeyer flasks and moistened with distilled water (20 g) to maintain a total moisture content of the solid waste of 80% (*5*). The flasks were autoclaved for 15 min at 103 kPa and 121 °C, cooled to room temperature and then five mycelial agar plugs of *A. sclerotiorum* PSU-RSPG178 were added to each flask. The flasks were incubated at 25 °C for 14 days. The obtained lovastatin yield was compared to that of SmF and the condition that gave the highest yield of lovastatin was selected for further experiments with other carbon sources.

### Supplementation with other sources

After the adjustment of moisture content, 10 mL of the supplements, comprising glucose 20 mg/mL, wheat flour 300 mg/mL, urea 2 mg/mL, molasses 0.03 mL/mL, palm oil and trace elements were added separately to the substrates, then incubated with five mycelial agar plugs of *A. sclerotiorum* at 25 °C for 14 days. The trace elements consisted of (in mg/mL): KH_2_PO_4_ 2, MgSO_4_ 0.3, CaCl_2_ 0.3, FeSO_4_ 0.11 and ZnSO_4_ 0.3 (*11*).

### Optimization of the lovastatin production

The best agricultural waste (soya bean sludge), cultivation process (SSF) and supplementary carbon source (palm oil) were selected from the above experiments to use in the optimization studies. The conditions were varied, including the amount of supplement (10, 20 or 30 mL of palm oil), the number of mycelial agar plugs (10, 20 or 30 plugs) and the cultivation time (14, 18 or 21 days).

### Analysis of moisture content

Samples were weighed before drying, then placed in a heat-resistant container which was heated at 135 °C for 2 hours, then weighed again to calculate the moisture mass fraction (*16*). The moisture content was calculated as the mass difference before and after drying and expressed as a mass fraction of the final dry mass of the sample. The analysis of moisture content was conducted in triplicate.

### Extraction and analysis of lovastatin

The lovastatin was extracted from the cells after cultivation according to the method of Daengrot *et al*. (*15*) and Phainuphong *et al*. (*13*). The fermented material was immersed in methanol for three days, followed by filtration through Whatman paper no. 0.5, then the methanol was evaporated with a rotary evaporator until the solution was viscous. Next, distilled water (50 mL) was added to the solution, and the mixture was extracted with hexane (2×100 mL). The hexane layer was separated and evaporated over anhydrous Na_2_SO_4_ under reduced pressure to obtain a crude extract in the form of a dark brown gum. The aqueous residue after extraction with hexane was further extracted twice with an equal amount of ethyl acetate (EtOAc). The EtOAc layer was dried over anhydrous Na_2_SO_4_ under reduced pressure to obtain a crude extract in the form of a dark brown gum.

### HPLC analysis

The crude extracts were transferred to a microcentrifuge tube and dissolved in 1.0 mL of acetonitrile. Lovastatin (98%, Acros Organics, Geel, Belgium) was used to prepare standard solutions in the same way as described for the sample solutions, containing 0, 40, 80, 160, 320 and 640 mg/L. Quantitative HPLC analyses were performed on an Agilent 1200 series DAD HPLC system using an ACE^®^ Generix 5 C18 column (250 mm×4.6 mm×5 mm i.d.). Acetonitrile was used as mobile phase A, and mobile phase B was aqueous 0.1% H_3_PO_4_ (Fisher Scientific). The flow rate was 1 mL/min, the injection volume 20 μL and peak detection was at 238 nm. Analysis started with 60% A and 40% B and lasted for 20 min (*13*).

### C/N analysis

The total carbon and nitrogen mass fraction (in %) in the fermented material were determined using a combustion method according to the AOAC method 993.13 (*17*) by a carbon to nitrogen determinator (CN628, LECO Corporation, St. Joseph, MI, USA) at the Central Equipment Division, Faculty of Science, Prince of Songkla University, Songkhla, Thailand.

### Statistical analysis

Statistical analysis was performed by one-way analysis of variance (ANOVA) and the Tukey’s *post*-*hoc* test with a significance level of p<0.05, using SPSS v. 17 (*18*). The data are presented as the mean value±S.D., with p<0.05 considered statistically significant.

## RESULTS AND DISCUSSION

### Screening of substrates for lovastatin production

Table 1 shows the initial moisture content of the substrates and the lovastatin yields obtained from them using SmF and SSF. Soya bean sludge had the highest moisture content (86%). The SmF system, using corn trunks as a substrate, showed the highest yield of lovastatin (0.004 mg/g) followed by soya bean sludge (0.002 mg/g), while *A. sclerotiorum* PSU-RSPG178 did not grow on the rice husks or on the wild sugarcane. Thus, corn trunks and soya bean sludge were used as substrates for SSF. In SSF with soya bean sludge and a 14-day cultivation period, the lovastatin yield was (0.040±0.002) mg/g. In the corn trunk medium, there was no growth after 14 days; however, there was growth after 30 days with a lovastatin yield of (0.164±0.001) mg/g. Although lovastatin production using corn trunks as the substrate was higher than that obtained using soya bean sludge, corn trunks require a size reduction step before use. Soya bean sludge has the advantage of being used directly. Additionally, it is plentiful, with about 1.7 million tonnes generated in Thailand during 2019 (*19*). Therefore, SSF using soya bean sludge was chosen for optimization of the other process conditions for lovastatin production.

**Table 1:** Lovastatin production, on dry mass basis, by *Aspergillus sclerotiorum* PSU-RSPG 178 with various substrates after 21 days of cultivation

Substrate	*w*(moisture)/%			*Y*(lovastatin)/(mg/g)			
Submerged fermentation		Solid-state fermentation	
Corn trunk		36.65±0.05	0.004±0.008		0.164±0.001	
Rice husk		3.92±0.08	NG		-	
Wild sugarcane		53.20±0.10	NG		-	
Soya bean sludge		86.00±0.02	0.002±0.001		0.037±0.002	

NG=no growth

### Effect of supplementation

**Fig. 1:**
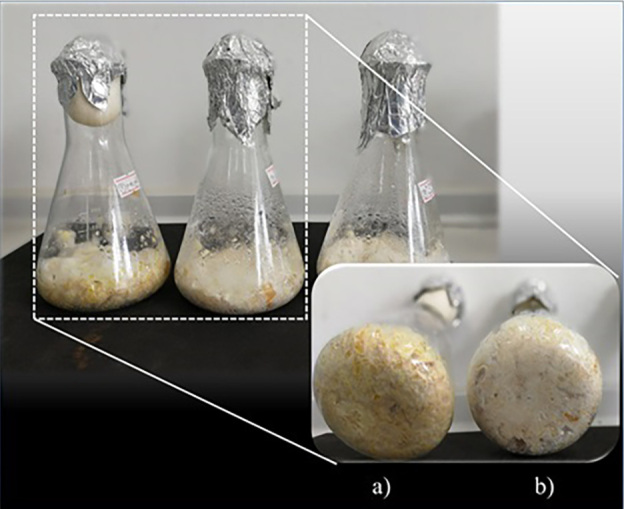
Characterization of *Aspergillus sclerotiorum* PSU-RSPG178 cultured in solid-state fermentation after 14 days on: a) soya bean sludge medium, and b) soya bean sludge medium supplemented with palm oil

Experiments were carried out in SSF with the soya bean sludge medium supplemented with other carbon sources. The soya bean sludge medium supplemented with palm oil gave the highest lovastatin yield (1.0 g/g) (Table 2). This lovastatin yield was almost 27-fold higher than the yield achieved in the control (without supplementation). At the end of the 14-day fermentation, the mycelium cultured on the soya bean sludge medium supplemented with palm oil was denser than on the culture without supplementation (Fig. 1). The mixture of residual solid substrate and fungal cells after lovastatin extraction was dried at 105 °C to a constant mass. The dry mass obtained for the soya bean sludge medium with or without palm oil was 47.5 and 9.7 g, respectively. These results agree with previous reports that vegetable oil improves lovastatin production (*20*, *21*).

**Table 2:** Yield of lovastatin on dry mass basis obtained from soya bean sludge with the addition of supplements

Type of culture medium	*Y*(lovastatin)/(mg/g)	
Soya bean sludge (control)		0.037±0.002
Soya bean sludge+10 mL glucose (*γ*=20 mg/mL)		0.096±0.002
Soya bean sludge+10 mL wheat flour (*γ*=0.3 g/mL)		0.057±0.001
Soya bean sludge+10 mL urea (*γ*=2 g/L)		0.025±0.001
Soya bean sludge+10 mL trace elements		0.022±0.001
Soya bean sludge+10 mL palm oil		0.992±0.003
Soya bean sludge+10 mL molasses (*φ*=0.03)		0.015±0.004

### Optimization of the lovastatin production

**Fig. 2:**
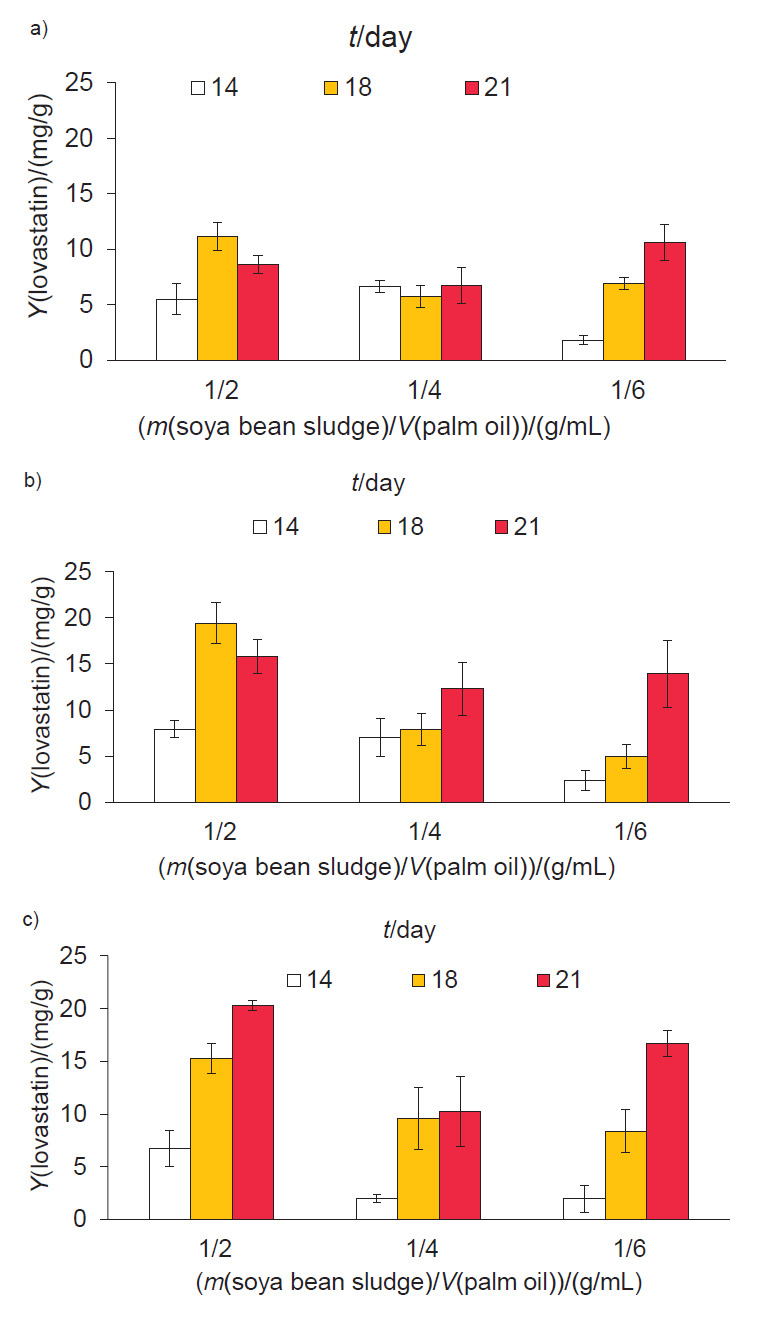
Lovastatin production by *Aspergillus sclerotiorum* PSU-RSPG178 using soya bean meal as a substrate in solid-state fermentation at different cultivation times and different ratios of soya bean sludge to palm oil. The ratio of soya bean sludge to mycelial agar plugs: a) 1:2, b) 1:4, and c) 1:6

Samples of dry soya bean sludge were maintained at an initial moisture content of 80% and then fermented for 14-21 days. The following parameters were varied: (*i*) the ratio of soya bean sludge mass (g) to palm oil (mL) from 1:2 to 1:6, and (*ii*) the ratio of soya bean sludge mass (g) to the number of mycelial agar plugs from 1:2 to 1:6. At a ratio of 1:2 soya bean sludge mass to mycelial agar plugs, where the ratio of soya bean sludge to palm oil was also 1:2, the highest lovastatin yield ((11±1) mg/g) was obtained after 18 days of cultivation (Fig. 2, a). At a ratio of soya bean sludge to mycelial agar plugs of 1:4 and to palm oil of 1:2, the highest lovastatin yield ((20±2) mg/g) was also obtained after 18 days of cultivation (Fig. 2b). At a ratio of soya bean sludge to mycelial agar plugs of 1:6 and to palm oil of 1:2, the highest lovastatin yield ((20±1) mg/g) was obtained after 21 days of cultivation (Fig. 2c). However, these last two were not significantly different (p>0.05).

The optimum conditions for lovastatin production were, therefore, a 1:2 ratio of soya bean sludge to palm oil, a 1:4 ratio of soya bean sludge to mycelial agar plugs and an 18-day cultivation period.The optimum ratio of soya bean sludge to palm oil of 1:2 corresponds to a C:N ratio of 42:1 (Table 3), which is similar to the optimum C:N ratio for lovastatin production of 40:1, found by López *et al*. (*22*) for the growth of *Aspergillus terreus* ATCC 20542 in SmF with lactose, in combination with either soybean meal or yeast extract under nitrogen-limited conditions.

**Table 3:** Initial total carbon and total nitrogen ratio of culture media

Culture medium	C:N
Soya bean sludge	9.85:1
Soya bean sludge to palm oil ratio: 1:2	41.87:1
1:4	62.76:1
1:6	84.25:1

**Fig. S1:**
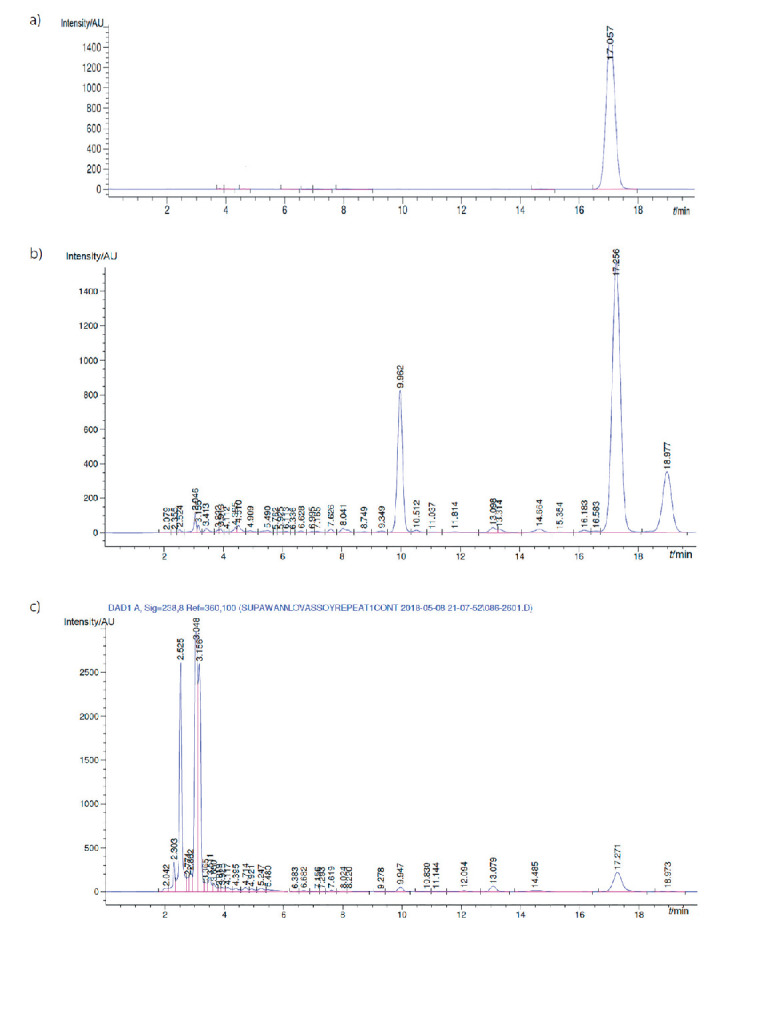
HPLC chromatograms of: a) lovastatin standard, and crude lovastatin extracts from: b) ethyl acetate and c) hexane

The HPLC chromatograms of the lovastatin obtained from this experiment compared with a lovastatin standard are shown in supplementary material (Fig. S1). The mass of the crude culture extracts obtained with ethyl acetate and hexane was 55.07 and 29.06 mg/g, respectively, and the lovastatin yields from ethyl acetate and hexane extract were 18 and 2 mg/g, respectively. Lovastatin is, therefore, more soluble in ethyl acetate than in hexane. The crude culture extracts obtained with ethyl acetate were further purified by column chromatography over silica gel using 2% MeOH/CH_2_Cl_2_ as eluent to obtain fourteen fractions. Fractions 5, 7 and 12 were lovastatin (*23*), penicillic acid (*24*) and monacolin S (*25*) respectively. These compounds were determined by comparison of the ^1^H NMR data with those previously reported.

**Fig. 3:**
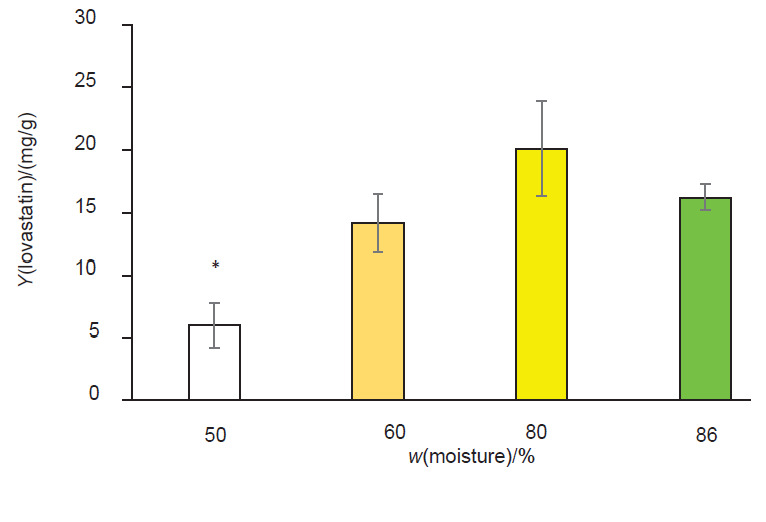
Lovastatin yields produced by *Aspergillus sclerotiorum* PSU-RSPG178 in a solid-state fermentation with different moisture contents. *represents significant difference at p<0.05

The obtained optimum condition was then tested at various moisture contents (50, 60, 80 and 86% after 18 days, with 86% moisture being that of fresh soya bean sludge. The mean values for lovastatin production were compared using Duncan’s new multiple range test (α=0.05). The lovastatin yield with a moisture mass fraction in soya bean sludge of 50% was significantly lower than when soya bean sludge with the higher moisture mass fractions was used (p<0.05) (Fig. 3). The lovastatin yields at 60, 80 and 86% of moisture in soya bean sludge were not significantly different (p>0.05). Therefore, fresh soya bean sludge can be directly used as a medium for lovastatin production without the need for drying and adjustment of moisture.

The lovastatin yields obtained from different fungal species grown on different media are quite difficult to compare and it is not a simple matter to determine which species is the best producer. In this study, *A. sclerotiorum* PSU-RSPG 178 produced (20±2) mg/g of lovastatin using soya bean sludge and palm oil as a medium, which is comparable with the maximal yield of lovastatin (20 mg/g) produced by *Monascus sanguineus* (Table 4 (*9*, *12*, *23*, *26*)). Although *M. sanguineus* produced lovastatin in a shorter time than *A. sclerotiorum* PSU-RSPG 178, it required a more complicated medium than *A. sclerotiorum* PSU-RSPG 178. An advantage of *A. sclerotiorum* PSU-RSPG 178 is that it also produces monacolin S, which has been found in red rice produced by *Monascus* spp., and has a more potent cholesterol-reducing effect than lovastatin (unpublished). Therefore, *A. sclerotiorum* PSU-RSPG 178 has a potential to produce a fermented food that has the same properties as red rice.

**Table 4:** Yield of lovastatin on dry mass basis obtained from other *Aspergillus* spp. and *Monascus* spp. under solid-state fermentation

*Aspergillus* spp. and *Monascus* spp.	Culture medium	Cultivation condition		*Y*(lovastatin)/ (mg/g)	Reference
*t*/°C	Time/day
*A. sclerotiorum* PSU-RSPG 178	Soya bean sludge	25	14	0.04	This work
	Soya bean sludge with palm oil	25	18	20.00	
*A. terreus* ATCC 74135	Rice straw	25	8	0.26	(*12*)
*A. terreus* ATCC 20542	Palm fronds, mineral and soybean meal	32	10	0.07	
*A. flavipes*	Soybean meal	30	6	0.75	(*9*)
*M. sanguineus*	Wheat bran with soybean 20 g/L, CaCl_2_ 2.5 g/L, acetic acid 25 μL	30 °C	16	20.04	(*23*)
Mixed seed cultures of *M. purpureus* and *M. ruber* (fungi for the production of red mould rice)	Long-grain, non-glutinous rice with malt extract 9.68 g/L, dextrose 38.90 g/L, MnSO_4_·H_2_O 1.96 g/L, and MgSO_4_·7H_2_O 0.730 g/L	29.46	13.89	2.80	(*26*)

## CONCLUSION

The highest lovastatin yield, (20±2) mg/g, was produced in 18 days of SSF with *Aspergillus sclerotiorum* using an agricultural waste, soya bean sludge, supplemented with palm oil. This yield was achieved with a ratio of 1 g soya bean sludge to 2 mL palm oil and inoculation with four mycelial agar plugs per g of soya bean sludge. Moreover, both fresh and dry soya bean sludge supplemented with palm oil can be used as alternative simple substrates. The present study also demonstrated that *A. sclerotiorum* could be used as a new candidate strain for lovastatin production.
